# Post-Operative Outcomes of Laparoscopic Appendectomy in Acute Complicated Appendicitis: A Single Center Study

**DOI:** 10.7759/cureus.38868

**Published:** 2023-05-11

**Authors:** Anurag Surabhi, Aparna Behura, Chinmay R Behera, Rajat K Patra, Bandita Panda, Amaresh Mishra, Ranjit Karnati, Subrat Mohanty

**Affiliations:** 1 Department of Surgery, Kalinga Institute of Medical Sciences, Bhubaneswar, IND; 2 Department of Pathology, Kalinga Institute of Medical Sciences, Bhubaneswar, IND; 3 Department of Surgery, Kalinga Institute of Medical sciences, Bhubaneswar, IND; 4 Department of Research and Development, Kalinga Institute of Medical Sciences, Bhubaneswar, IND; 5 Department of Surgery (Pediatric Surgery), Kalinga Institute of Medical Sciences, Bhubaneswar, IND

**Keywords:** intra abdominal abscess, appendicitis, post-operative outcome, laparoscopic appendectomy, acute complicated appendicitis

## Abstract

Background: Acute appendicitis (AA) is a surgical emergency because of inflammation in the appendix leading to swelling, whereas acute complicated appendicitis is characterized by a gangrenous or perforated appendix with or without periappendicular abscess, peritonitis, and an appendicular mass. The laparoscopic approach in complicated acute appendicitis is a viable alternative method but is not practiced in all cases because of technical difficulties and unpredictable complications. Thus, the present study aimed to evaluate the primary and secondary outcome predictors of laparoscopic appendectomy in complicated appendicitis.

Methods: A single-center prospective observational study was carried out after the approval of the Institutional Ethics Committee (IEC). A total of 87 complicated acute appendicitis patients were included in the study. Clinico-demographic features such as age, gender, duration of surgery, post-operative pain, and hospital stay were monitored in different age groups of <20, 20-39, and >40 years, and the primary and secondary outcomes of laparoscopic surgery in acute complicated appendicitis were measured.

Result: Acute complicated appendicitis cases were observed mostly in people older than 42 years in the total study population. Laparoscopic appendectomy was conducted in all 87 acute complicated appendicitis patients, and the major surgical outcome predictors were monitored, such as mean operating time (87.9 minutes), post-operative pain (3.9 scores), and post-operative stay (6.7 days). Post-operative complications such as drain site infection (1.14%), enterocutaneous fistula (2%), and intra-abdominal abscess (7%) were observed.

Conclusion: Based on our observations, a laparoscopic appendectomy can be considered a viable alternative with an acceptable complication rate. Operative time varies from 84 to 94 minutes in different age groups and with the extent of the disease.

## Introduction

Acute appendicitis is a surgical emergency reported with an incidence of 8%, but the incidence of acute complicated appendicitis may be higher [1,2]. Laparoscopic appendectomy has the advantage of better peritoneal cavity visibility with minimal access, even in complicated cases with periappendicular adhesions, mass and abscess formation, and abdominal distension. Sometimes it may require more expertise and longer operative time. However, in this setting, wound surface contamination is less than in open access. The development of intra-abdominal abscesses is an independent risk factor.

In spite of laparoscopic appendectomy being technically challenging in acute complicated appendicitis and some studies showing greater post-operative morbidity, in recent years it has become the preferred option of treatment rather than an open procedure.

Because of the prolonged time, high risk of post-operative intra-abdominal abscess, and increased procedural costs, managing acute complicated appendicitis by laparoscopic appendectomy has not yet been considered a gold standard procedure. Thus, the present study aimed to evaluate the feasibility of the laparoscopic approach in acute complicated appendicitis by assessing the operative time, post-operative complications, and duration of hospital stay.

## Materials and methods

A hospital-based prospective observational study was conducted at the surgery department of a tertiary care centre, Kalinga Institute of Medical Sciences, Bhubaneswar, during the period of two years (2020-2022). After the approval of the institutional ethics committee (Ref. No. KIIT/KIMS/484/2020) and the patient’s consent, the study recruited consecutive acutely complicated appendicitis patients based on the inclusion and exclusion criteria. Major complications included as per the definition are periappendicular adhesions, masses, abscess formation, and abdominal distension. Clinical diagnosis was done through physical examination and radiological imaging. The previous history of any abdominal surgeries was excluded.

**Figure 1:**
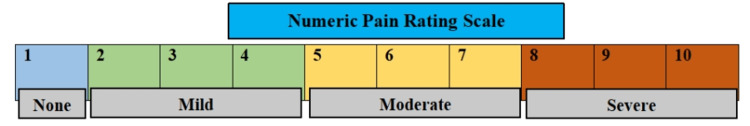
Numeric pain rating scale Adapted from McCaffery and Beebe [3]

A total of 87 patients admitted consecutively during the study period were included. A detailed history with demographic features and clinical and radiological examinations was recorded. Clinical outcomes, surgical duration, and secondary outcomes such as post-operative pain, the occurrence of post-operative complications, and length of hospital stay were evaluated. Post-operative pain was measured using the Numeric Pain Rating Scale Instructions. [3] The pain levels were monitored for 24 hours and scored on a scale of 0 to 10. The average of the 3 ratings was used as the patient’s level of pain (Figure 1).

Demographic features such as age, gender, duration of surgery, post-operative pain, and hospital stay were monitored in different age groups. All the patients were categorized according to the three age groups such as below 20 years, 20-39 years, and above 40 years. The duration of surgery, post-operative pain, and post-operative complications were monitored in different age groups (<20, 20-39, and >40 years) of patients.

Statistical analysis

The mean and standard deviation were applied to all the continuous data, and comparative evaluation was done by student t-test. All categorical data were presented in frequencies and percentages. Significance levels between the different age groups were measured by t-test, and a p-value ≤ 0.05 was used as statistically significant. Mann-Whitney U test was used to measure the central tendency of outcome variables between the two groups. All the statistical calculations were done using SPSS software version 25 (IBM Corp., Armonk, NY).

## Results

**Figure 2:**
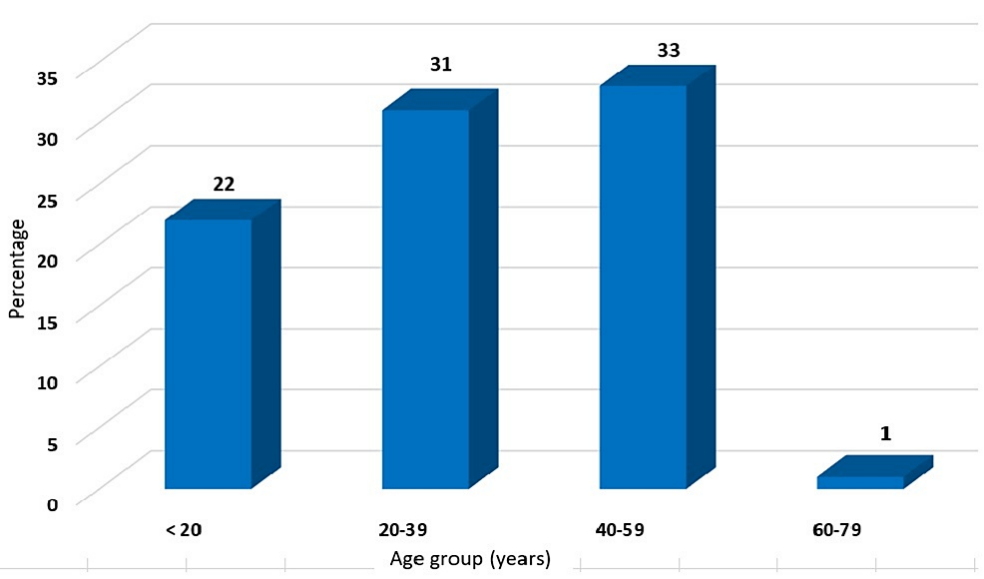
Distribution of appendicitis patients underwent for laparoscopic appendectomy as per the age group (<20, 20-39, 40-59, 60-79 years)

A total of 87 patients underwent a laparoscopic appendectomy. Demographic features and surgical outcomes of all patients were recorded, and the findings are presented in Table 1. The mean age was 32.89 ± 13.93 years, and all the patients were categorized into four groups such as less than 20, 20-39, 40-59, and 60-79 years. The frequency of patients in less than 20 years was 25.28%; in the 20-39 year age group, it was 35.63%; and in the 40-59 year age group, the frequency was 37.93% (Figure 2). In gender distribution, the male-female ratio was 2:1 (66.7% male: 33.3% female).

**Table 1:** Clinico-demographic features of acute complicated appendicitis undergone for laparoscopic appendectomy

Clinico-Demographic features	N=87
Age (mean ± SD) years	32.89 ± 13.93
Gender (frequency in percentage)	Female: 33.3%; male: 66.7%
Post-operative time (minutes mean ± SD) mins	87.93 ± 30.68
Post-operative pain (mean ± SD)	3.62 ± 1.85
Hospital stay (mean ± SD) days	6.7 ± 23.95

In the surgical and post-surgery outcomes of laparoscopic appendectomies, such as operation time, post-operative pain, and hospital stay, all patients were recorded. Operative time varied from patient to patient, and the range is from 33 minutes to 201 minutes (3 hours, 20 minutes), with a mean operating time of 87.93 minutes ± 30.68 minutes. The operative time was measured from skin incision to skin stapling. The different age groups with an operative time are shown in Table 2, revealing that in less than 20 years the operative time was maximum (94.68 minutes ± 31.911) as compared to the other two groups (20-39 and above 40 years), but statistically, no significant difference (p-value = 0.490) was observed between the age groups. Post-operative pain was measured for every patient 24 hours post-surgery; some patients were found to be painless, and the majority had minimal to moderate pain. The pain was measured using the Numeric Pain Rating Scale Instructions (adapted from McCaffery and Beebe [3]). The mean score for post-operative pain was 3.62 ± 1.85. In three different age groups, the mean pain score in less than 20-year-old patients was 3.5 ± 1.76, whereas in the age groups of 20-39 and above 40 years, the mean pain score was 3.94 ± 1.96 and 3.41 ± 1.84, respectively. The statistical variation between the groups was insignificant (p-value: 0.501). The mean hospital stay for all patients was six days, as shown in Table 2. In different age groups, it was observed that in the less than 20-year-old age group, the hospital stay was 8.82 days, whereas in the 20-39-year-old age group, it was 6.16 days, and in the more than 40-year-old age group, the hospital stay was five days (Table 2). The statistical variation in hospital stay between the groups was observed, and it was significantly less (p-value: 0.014) in 40 years and above than in <20 and 20-39 age groups.

**Table 2:** The post-operative outcome of laparoscopic appendectomy of different age groups (years)

Outcome	Age group	Mean ± SD (N=87)	p-value
Operation time (in minutes)	<20	94.68 ± 31.911	0.490 (NS)
	20–39	86.45 ± 29.517	
	40 and above	84.91 ± 31.62	
Post-operative pain	<20	3.5 ± 1.76	0.501 (NS)
	20–39	3.94 ± 1.96	
	40 and above	3.41 ± 1.84	
Hospital stay (in days)	<20	8.82 ± 4.6	0.014 (S)
	20–39	6.16 ± 4.1	
	40 and above	5.88 ± 2.8	

**Figure 3:**
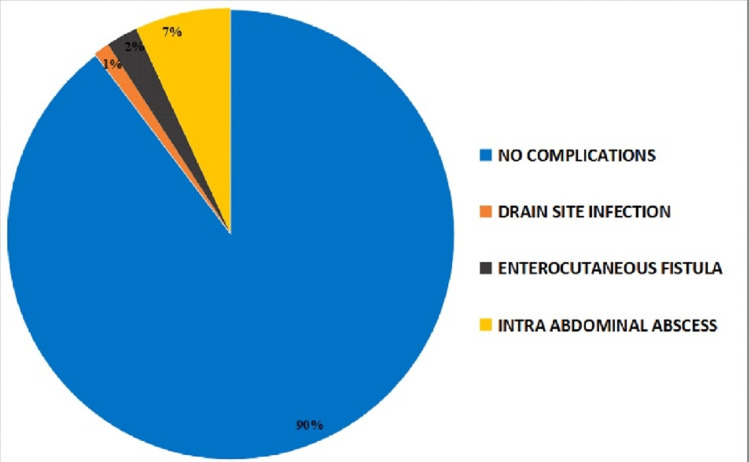
Complications observed in laparoscopic appendectomy of appendicitis patients

Complications were minutely observed in all patients. Among the 87 patients, nine (10.3%) were found to have complications (Figure 3). Complications such as drain site infection, enterocutaneous fistula, and intra-abdominal abscess were observed in 1%, 2%, and 7% of patients, respectively, which were successfully managed clinically.

## Discussion

Strong evidence-based data are limited in the surgical management of complicated appendicitis by LA. Wullstein et al. reported a case series on the advantages of minimally invasive techniques in complicated appendicitis [4]. In comparison between appendicitis with no complication and complicated appendicitis, post-operative factors such as analgesia requirements, length of hospital stay, return to regular activity intervals, and complication rates are debatable in the case of complicated appendicitis. Complicated appendicitis is associated with a higher risk of post-operative complications and has been considered a relative contraindication for laparoscopy [5-7]. However, this concept has been challenged in some studies that compared the surgical outcomes of LA for complicated appendicitis [8-10].

**Figure 4:**
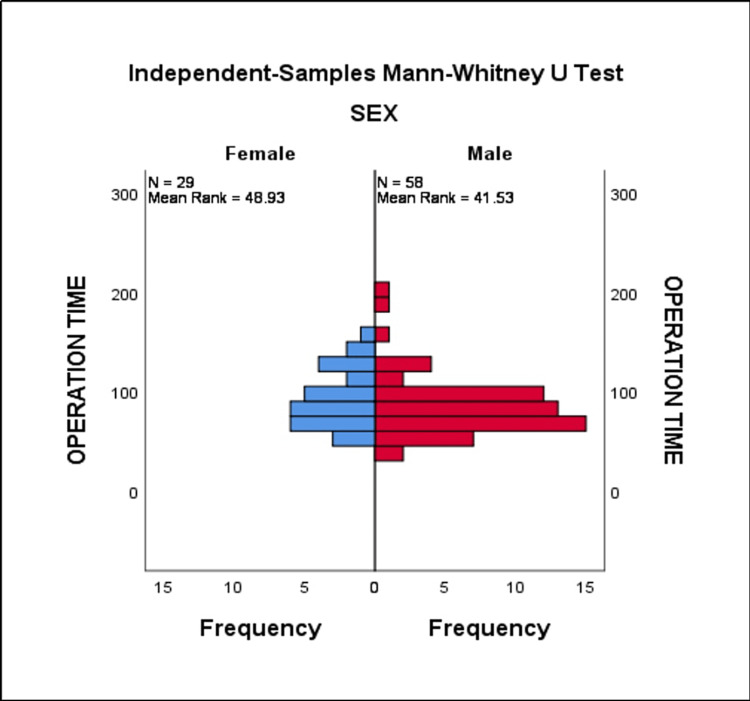
Mean rank of operation time in both male and female by Mann-Whitney U test

The present study evaluates laparoscopic appendectomy in different age groups. In comparison with different age groups (below 20 years, 20-39 years, and above 40 years), most of the patients were presented in the age group above 40 years, followed by the 20-30 year age group. This finding is supported by the results of Lasek et al. [11]. Similar results were reported in another study of 69 cases operated through LA [12]. In terms of gender distribution, the incidence of acute complicated appendicitis was observed to be higher in males than females and at its maximum in the age group of 40-59 years (Figure 4).

The mean operating time in the total study population was 87.9 minutes, and in comparison to different age groups, the operating time was observed to be higher in the below-20-year-old age group, followed by the 20-39-year-old age group, because of time consumption for peritoneal lavage and tying of the base. It has been observed that as age increased, the operating time decreased. Many studies have reported that the operating time in LA is longer compared to an open appendectomy [13-15]. However, Yau et al. reported that the operative time is less in laparoscopic appendectomy than in open appendectomy [16].

In LA, the analgesic requirements and post-operative pain are less as compared to open surgery [17-21]. This agrees with the current study, where the average post-operative pain is 3.9 according to the Numeric Pain Rating Scale Instructions (adapted from McCaffery and Beebe [3]). In comparison to different age groups, post-operative pain was more common in the 20-39 age group.

The duration of drainage and hospital stays was low in LA. The average post-operative stay was 6.7 days. Like operating time, the hospital stay was also higher in the below-20-year-old age group than in more than 40-year-old age groups, as well as in 20-39-year-old age groups. These results were comparable to many previous studies. Additionally, there were some reports that explained that oral intake is quite early in LA compared to OA but statistically insignificant [17,18].

Post-operative complications in LA

Complications encountered in this study are drain site infection (1.14%), enterocutaneous fistula (2%), and intra-abdominal abscess (7%). The rest 90% of cases were free from post-operative complications. The occurrence of wound infections was lower, which is one of the major advantages of LA. In our study, post-operative wound infections were observed in 1.14% of the total LA study population because of the zero contact point in trocar wounds and the removal of the appendix in a disposable bag. The infected fluid was removed by proper aspiration through the laparoscope. In open appendicitis, the wound infection is higher due to direct contact of the abdominal incision with both the appendix and infected fluid. Similar results were demonstrated in other studies as well [22]. In our study, the incidence of intra-abdominal abscesses was lower (6.9%), and the result supports Temple et al., Krukowski et al., and Reid et al. [23-25]. Sufficient pre-operative resuscitation, appropriate peri-operative antibiotics, and the use of standardized surgical techniques are attributed to the lower incidence of abdominal abscesses. Though faecal fistula is an abnormal passage that communicates with the intestine, post-appendectomy faecal fistula formation is a complication and is associated with significant morbidity [26]. When there is severe periappendicitis, post-appendectomy faecal fistulas mostly occur, which involve the base of the appendix as well as the adjoining faecal wall. In such cases, the major aetiological factor, leakage from the appendiceal stump, is incriminated, as are injuries to the caecum [27]. Post-appendectomy faecal fistulas are reported by Genier et al. by reviewing 22 cases in a 24-year period, of which 21 were severe (suppurative, gangrenous, or perforated) [28]. The limitation of the study is the inadequate sample size for a significant outcome on operation time, post-operative pain, etc.

## Conclusions

Laparoscopic appendectomy for complicated acute appendicitis is not always the preferred choice of many surgeons. Based on the zero conversion rate, limited post-surgery complications, and mild post-operative pain, the present study concluded that laparoscopic appendectomy can be considered a safe, feasible, and first-choice surgical approach. Our study suggests that operation time, post-operative pain, and hospital stay are comparatively lesser in the age group of 40-50 years. In our study, the operative time varied from 84 to 94 minutes because of the different age groups and the extent of the complications.
